# Comparative phenotypic and transcriptomic analyses reveal the potential molecular basis of forming bigger leaf blade in autotetraploid castor bean

**DOI:** 10.1186/s12870-025-07229-8

**Published:** 2025-09-25

**Authors:** Jian Wu, Wei Shu, Yanyu Zhang, Dan He, Bing Han, Anmin Yu, Qinghua Cui, Aizhong Liu

**Affiliations:** 1 Key Laboratory for Forest Resources Conservation and Utilization in the Southwest Mountains of China, Ministry of Education, Southwest Forestry University, Kunming, 650224 China; 2 School of Life Sciences, Yunnan University, Kunming, 650091 China; 3 Germplasm Bank of Wild Species, Kunming Institute of Botany, Chinese Academy of Sciences, Kunming, 650201 China

**Keywords:** Castor bean, Autotetraploid, Leaf size, Cell expansion, Cell division, Plant hormone

## Abstract

**Background:**

Whole-genome duplication events often confer autopolyploid plants with bigger leaf blades compared with those of their diploid counterparts. However, little is known regarding the potential molecular basis of bigger leaf formation in autopolyploid plants. Here, we focused on the oilseed crop castor bean (*Ricinus communis L.*) to investigate the molecular basis underlying leaf size variation using a synthetic autotetraploid by doubling the diploid homologous chromosomes.

**Results:**

The results showed that the leaf area of autotetraploids was significantly larger than that of diploids. According to our histological observations, the formation of larger leaf blades in tetraploid castor beans is attributed to both an increase in cell size and an increase in cell number. A total of 3,464 differentially expressed genes (DEGs) between diploids and tetraploids were identified by RNA sequencing analysis. The expression of key genes related to cell wall loosening, cell expansion and cell division was higher in tetraploid leaf blades compared to diploids, resulting in enlarged tetraploid leaf blades, such as *SUS2*, *SUS4*, *XYL1*, *Xyl2*, *XTH30*,* XTH32*,* EXPA1*, *EXPA4*, *EXPA6*, *EXPB3*, *CYCD3*;*1* and *CYCD3*;*3* were significantly up-regulated in tetraploids. Concurrently, auxin-responsive genes (*SAUR20*, *SAUR23*, and *SAUR51*) in the auxin signaling pathway showed significant up-regulated in tetraploids, facilitating leaf cell expansion. Transcription factors (TFs) including *HAT22*, *SRM1*, *ERF4*, and *DOF3.4* likely regulate cell expansion and elongation pathways, ultimately driving the enlargement of tetraploid leaf blades.

**Conclusions:**

Our findings provide important insight into understanding the potential molecular basis of gene dosage effects on trait variation in autopolyploid plants.

**Supplementary Information:**

The online version contains supplementary material available at 10.1186/s12870-025-07229-8.

## Background

Polyploid plants, with three or more sets of homologous chromosomes, have long been recognized as a pivotal driver in angiosperm evolution [1, 2]. Polyploids were traditionally classified into autopolyploids (multiplications of a single genome) and allopolyploids (combinations of two or more divergent genomes) according to the origin of the chromosome sets [3, 4]. Many economically important crops in agriculture and forestry are polyploids, such as *Prunus pseudocerasus* [5], *Pogostemon cablin* [6], *Triticum aestivum* [7], *Rhododendron fortunei* [8], and *Robinia* [9, 10]. Polyploidization has played a critical role in plant speciation and crop evolution [11–13]. Allopolyploids are generally thought to have several evolutionary advantages over autopolyploids [4, 14, 15], including the fixation of both heterozygosity and genetic redundancy in allopolyploids, which facilitates the evolution of new traits and increases adaptability. However, an increasing number of studies have suggested that the true occurrence of autopolyploids may be underestimated [16–19]. Autopolyploidy might play an indispensable role in plant diversification and speciation.

The increase in chromosome number in polyploids is attributed to whole-genome duplication events [11, 20, 21]. Whole-genome duplication, to a large extent, enhances genomic plasticity for functional differentiation of duplicated genes, genome restructuring, and transcriptome changes, hence contributing to evolution [1, 21–24]. However, how whole-genome duplication affects plant growth and development, and reshapes genetic traits, largely remains unanswered. Previous attempts to unravel the mechanisms underlying the effects of whole-genome duplication on phenotypic variation were mainly focused on allopolyploids [4, 14, 25]. Nevertheless, allopolyploids are derived from hybridization of distinct parental genomes; a significant portion of genetic variation may be caused by the merging of two or more diverged genomes [3]. To avoid this drawback, interests have shifted toward autopolyploids, which originate from the multiplication of a diploid genome and only exhibit gene dosage effects [3, 16]. Consequently, autopolyploids, particularly synthetic lines, provide ideal experimental systems for investigating the phenotypic consequences of whole-genome duplication, owing to their uniform genetic backgrounds.

In higher plants, one of the most intriguing consequences of homologous chromosome doubling is the increased dosage of all genes, which may increase the size of cells and organelles by altering gene expression, ultimately leading to phenotypic variation of plants [26–28]. Thus, autopolyploid plants often show prominent vegetative growth advantages compared with those of their isogenic diploid counterparts, such as *Brassica rapa* [29], *Citrullus lanatus* [30], *Oryza sativa* [27], *Mangifera indica* [19], and *Arabidopsis* [31]. These growth advantages, including but not limited to larger leaf blades [32–34], more vigorous growth [35], thicker stems [36, 37], larger flowers [38, 39], and greater seed size [27, 40], are often observed in synthetic autotetraploid plants. Although these studies have provided important information in understanding the effects of whole-genome duplication on phenotype variation, little is known about the cellular and molecular mechanisms underlying the leaf size superiority of autopolyploid plants.

Castor bean (*Ricinus communis L.*) is, an economically important non-edible oilseed crop, widely cultivated in many countries and regions worldwide, particularly India, Brazil and China [41]. Since its seed oils are mainly composed of unique ricinoleic acid (a kind of hydroxy fatty acid with a hydroxyl attached to C12). Castor oils have been widely used in industry such as lubricants, nylon, adhesive, aviation oil, furniture coating and feedstock of biodiesel [42, 43]. Owing to the high economic value of castor oils, breeding and genetic improvement of castor bean varieties are drawing broad attention from crop breeders. Since adequate vegetative growth is essential to achieve high seed yield in castor bean, dissecting the molecular basis of fast growth and yield traits is critical for developing genetically improved varieties.

Here, we characterized the molecular basis of leaf size variation following homologous chromosome doubling in castor bean. To this end, we used diploid variety ZB306 and its synthetic autotetraploid to analyze mechanisms of leaf enlargement in autotetraploid plants. Combining with comparative transcription analysis, we identified diverse differentially expressed genes (DEGs) and transcription factors (TFs) that were specifically involved in leaf cell growth. These findings reveal molecular mechanisms underlying enlarged tetraploid leaves, informing strategies for trait improvement in castor bean breeding.

## Methods

### Plant materials

Seeds of the diploid and autotetraploid castor bean variety (ZB306) were supplied from the Kunming Institute of Botany, Chinese Academy of Sciences, Kunming, China. Among them, autopolyploids were obtained through 0.5% colchicine treatment of diploid seeds. Seeds of both the diploid and autotetraploid were initially germinated on water-soaked filter paper for 2 days and then transplanted into plastic pots containing a 1:1:2 mixture of turfy soil, perlite, and sand. All plant materials were grown in the greenhouse at Southwest Forestry University, Kunming, China.

### Ploidy level determination and sample preparation

The ploidy levels of all plantlets derived from seeds were initially assessed using flow cytometry analysis (Partec-PAS, Germany) according to the previously reported [44]. *Zea mays* variety (B73) was used as an internal reference standard for flow cytometry analysis. Subsequently, the putative tetraploid plants were definitively confirmed through somatic chromosome counting as previously described methodology [45]. Following ploidy level confirmation, 15 diploid and 15 tetraploid rooted plantlets with prolific root systems were sampled and transplanted into the field. After three months of cultivation, the images of the mature leaf blades were recorded by a digital camera, and the leaf area was measured by ImageJ software (http://imagej.net/ij/). Mature leaf samples from both diploid and tetraploid plants were collected for stomatal and cross-sectional cellular observations. Subsequently, young leaves (third or fourth position from the apex) were rapidly excised from diploid and tetraploid plants, immediately frozen in liquid nitrogen, and stored in a freezer at −80 °C for subsequent analyses, including phytohormone quantification, RNA sequencing (RNA-seq), and quantitative real-time PCR (qRT-PCR) validation. All experiments were conducted with three independent biological replicates.

### Analysis of abaxial epidermal cell and stomatal characteristics

Fully expanded leaves from six plantlets (three diploids and three tetraploids) were collected for epidermal and stomatal characterization. Leaf samples containing main veins were fixed for 24 h in FAA solution (38% formaldehyde : glacial acetic acid : 70% ethanol = 1 : 1 : 18, v/v/v). Following fixation, samples were rinsed three times with distilled water and dried on filter paper. Epidermal and stomatal observations were conducted using an Olympus BX51 microscope (Olympus, Tokyo, Japan) equipped with a 20× oil immersion objective. Stomatal dimensions (length and width) were measured for 30 randomly selected stomata per sample.

### Histochemical staining

The cross-sectional cellular characteristics of leaf veins in diploid and tetraploid plants were analyzed following previously described methods [46]. Fixed leaf samples were dehydrated through an ethanol gradient series (70%, 80%, and 100% ethanol; 1 h per concentration), followed by sequential treatment with ethanol–dimethylbenzene solution (1:1, v/v) for 30 min and pure dimethylbenzene for an additional 30 min. Samples were then infiltrated with paraffin through sequential incubations in paraffin-dimethylbenzene mixtures (1:1, v/v) at 60 °C for 6 h, followed by three times of pure paraffin (60 °C, 2 h each time). Subsequently, each sample was embedded in a paper cup for solidification. The paraffin blocks were trimmed to appropriate dimensions and sectioned at 8 μm thickness using a rotary microtome. After dewaxing, the paraffin sections were sequentially stained with 1% safranin O and counterstained with 0.1% fast green. Microscopic observation and image acquisition were performed using an Olympus BX51 microscope.

### Extraction and analysis of endogenous hormones

Leaf samples were ground to a fine powder in liquid nitrogen with a mortar and pestle, and suspended in potassium phosphate buffer (50 mM, pH = 7.4). After vigorous shaking, they were centrifuged for 30 min at 8000 rpm at 4 °C. The total content of endogenous hormones, including indole-3-acetic acid (IAA), gibberellins (GA), brassinosteroids (BR), and biologically active cytokinin (CTK), was determined using enzyme-linked immunosorbent assay (ELISA) kits (Shanghai Enzyme-linked Biotechnology Co., Ltd., China) according to the manufacturer’s instructions. Three independent biological replicates were established for each ploidy level, with triplicate technical replicates for each biological replicate. All statistical analyses were implemented using IBM SPSS Statistics software (version 20.0; IBM Inc., New York, USA). Independent two-sample *t*-tests were performed to assess significant differences between diploid and tetraploid plants.

### RNA-Seq library construction and sequencing

The young leaf samples of three-month-old diploid and tetraploid plants were collected for RNA-seq, with the diploid material denoted as D1, D2, and D3 and the tetraploid material denoted as T1, T2, and T3, respectively. Total RNA was isolated from these samples using TRIzol Reagent Kits (Invitrogen, Carlsbad, CA, USA), followed by using a NanoDrop 2000 bioanalyzer (Thermo Fisher Scientific Inc., Wilmington, DE, USA) to determine the quality. The RNA integrity was evaluated using the RNA Nano 6000 Assay Kit on an Agilent Bioanalyzer 2100 system (Agilent Technologies, Santa Clara, CA, USA). Approximately 3 µg of total RNA from each sample served as the starting material for cDNA library construction. Six cDNA libraries were amplified and prepared for sequencing. High-throughput RNA sequencing was performed on an Illumina NovaSeq 6000 sequencing platform (Illumina, San Diego, CA, USA), generating 150 bp paired-end reads.

### Bioinformatics analysis of RNA-Seq data

To obtain high-quality clean data for further bioinformatics analysis, raw sequencing reads were processed using Fastp software (version 0.12) to remove adapter sequences, poly-N-containing reads, and low-quality sequences. Meanwhile, sequencing quality was assessed by calculating Q20, Q30 scores, GC content, and sequence duplication levels from the filtered reads. High-quality clean reads were then mapped to the castor bean reference genome using HISAT2 software [47], and mapping rates were determined. Gene expression levels were quantified using featureCounts software, and Fragments per kilobase of transcript per million fragments mapped reads (FPKM) values were calculated to normalize gene expression across samples [48].

### Identification of DEGs and functional analysis

Identification of the DEGs in diploid and tetraploid plants was conducted using the DESeq2 package [49] with stringent thresholds: a false discovery rate (FDR) < 0.05 and |log_2_(fold change)| ≥ 1. To identify the specific function of DEGs in diploids and tetraploids, the Wallenius’ non-central hypergeometric distribution and GOseq R package was used to implement the GO (http://geneontology.org/) enrichment analysis [50]. Additionally, the DEGs enriched in KEGG (http://www.kegg.jp/) pathways were analyzed using the KOBAS 2.0 software [51]. GO terms and KEGG pathways were considered significantly enriched when *p*-adjust value < 0.05. TFs from DEGs were predicted using the PlantTFDB (https://planttfdb.gao-lab.org/).

### Gene co-expression network analysis and visualization

Gene expression levels quantified as FPKM were used for co-expression analysis. Pairwise Pearson correlation coefficients between gene expression profiles were calculated using the R package. The screening threshold was |*r*| *≥* 0.8 and *p* < 0.05, where positive and negative values indicated positive and negative correlations, respectively. Gene co-expression networks were visualized using Cytoscape software (version 3.9.0), which was also used to calculate gene connectivity degrees. The node size was positively correlated with the degree of the connectivity of the genes.

### Quantitative RT–PCR validation and expression analysis

Total RNA extracted from the leaf samples of diploid and tetraploid plants was served as a template for cDNA synthesis and reverse-transcribed using the TransScript All-in-One First-Strand cDNA Synthesis SuperMix for qPCR kit (TransGen Biotech, Beijing, China). The qRT-PCR test was conducted using PerfectStart Green qPCR SuperMix (TransGen Biotech, Beijing, China) according to the manufacturer’s recommendations with the Bio-Rad CFX96 system (California, USA). The sequences of the primers were designed using an online tool (http://www.ncbi.nlm.nih.gov/tools/primer-blast/). A castor bean ACTIN2 gene was utilized as the reference gene and amplified in parallel with target genes, allowing gene expression normalization. All primer sequences in this experiment are provided in Table S1. Relative gene expression levels were calculated using the 2^−∆∆CT^ method. A total of eight DEGs were selected for qRT-PCR validation, with triplicate technical replicates and three independent biological replicates performed for each gene.

## Results

### Ploidy level determination and morphological variation analysis

**Fig. 1:**
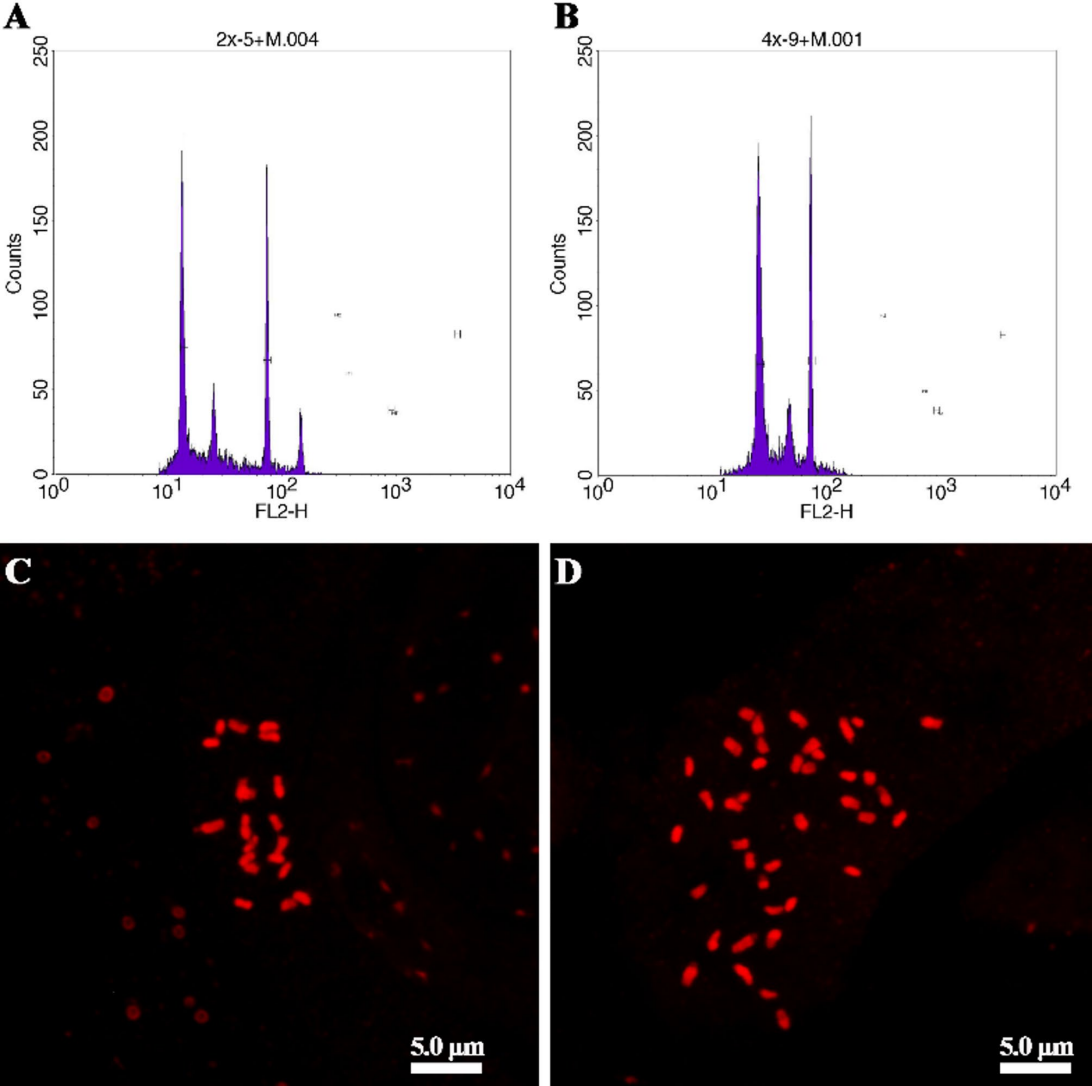
Determination of the ploidy levels of castor bean plants. **A** Flow cytometry analysis of the diploids. **B** Flow cytometry analysis of the tetraploids. **C** Somatic chromosome number of the diploids (2n = 2*x* = 20). (D) Somatic chromosome number of the tetraploids (2n = 4*x* = 40)

To confirm the ploidy level of all plantlets germinating from castor bean seeds, we identified diploids and true tetraploids by flow cytometry analysis and somatic chromosome counting. As shown in Fig. 1, individual castor bean plants were preliminarily classified as diploid (Fig. 1A) or tetraploid (Fig. 1B) based on the peaks obtained by flow cytometry analysis. The ploidy level of diploids and tetraploids was ultimately confirmed by somatic chromosome counting. Somatic chromosome number of diploids was 2n = 2*x* = 20 (Fig. 1C), and the somatic chromosome number of tetraploids was 2n = 4*x* = 40 (Fig. 1D).

**Fig. 2:**
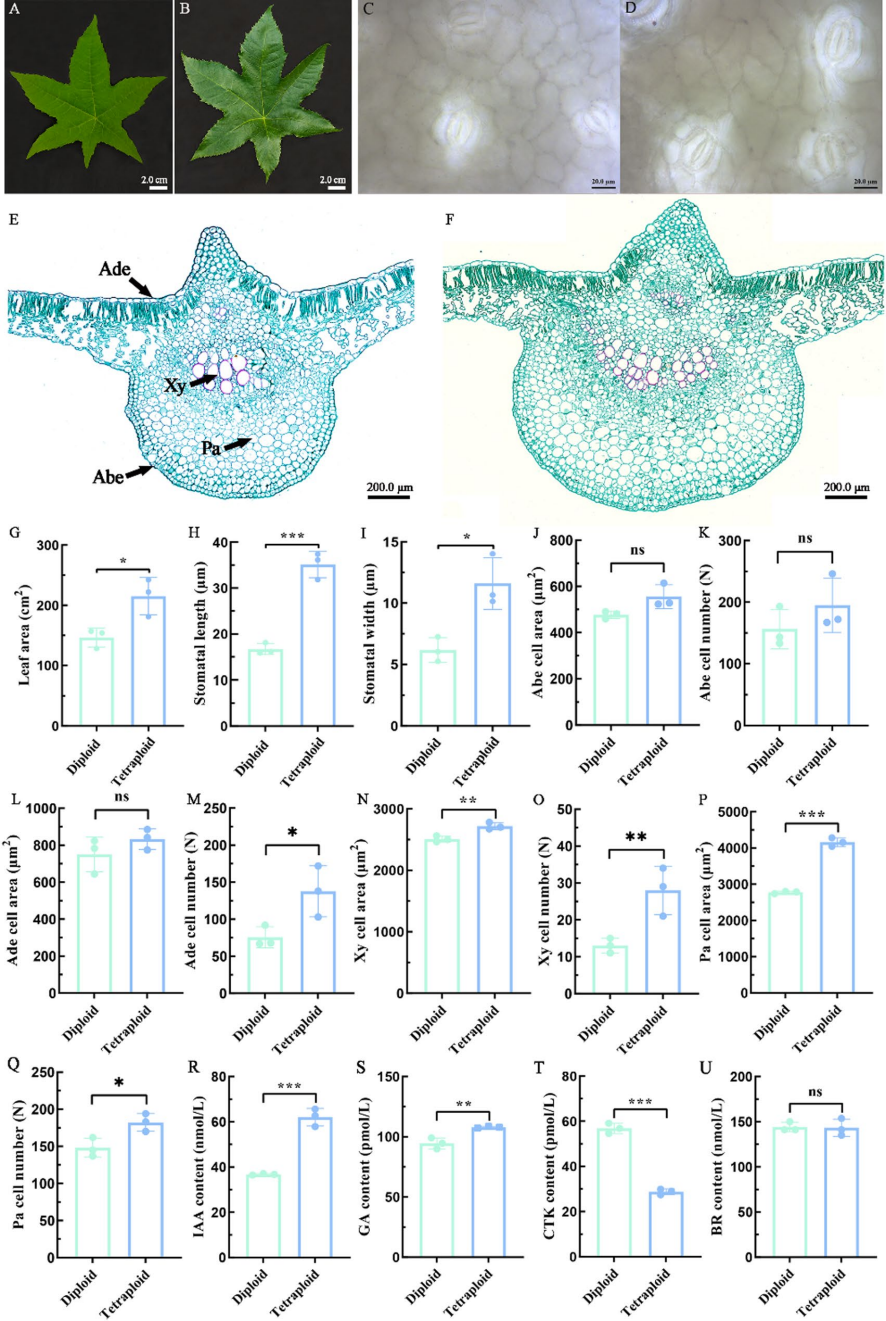
Morphological changes in diploid and tetraploid leaf blades. Fully expanded leaf blades in diploid (**A**) and tetraploid (**B**) plants. Epidermal stomatal characteristics of the leaf blades in diploid (**C**) and tetraploid (**D**) plants. The cross-sectional cell of leaf veins in diploid (**E**) and tetraploid (**F**) plants. The leaf area (**G**), stomatal length (**H**), stomatal width (**I**), Abe cell area (**J**), Abe cell number (**K**), Ade cell area (**L**), Ade cell number (**M**), Xy cell area (**N**), Xy cell number (**O**), Pa cell area (**P**), Pa cell number (**Q**), IAA content (**R**), GA content (**S**), CTK content (**T**), BR content (**U**) in diploid and tetraploid plants. Ade: adaxial epidermis; Abe: abaxial epidermis; Xy: xylem; Pa: parenchyma. The vertical bars show the standard error; the asterisk indicates significant differences between diploid and tetraploid plants (^ns^ *p* > 0.05, * *p* < 0.05, ** *p* < 0.01, *** *p* < 0.001)

To evaluate the impact of ploidy level on phenotypic variation, 15 diploid and 15 tetraploid plants were transplanted and cultivated under field conditions for three months. Comparative analysis of leaf morphological traits revealed significant differences between ploidy levels. Tetraploid plants displayed substantial increases in leaf blade size (Fig. 2A, B) and stomatal dimensions (Fig. 2C, D) compared to their diploid counterparts. Further measurements demonstrated that tetraploids exhibited significantly greater leaf area (Fig. 2G), stomatal length (Fig. 2H), and stomatal width (Fig. 2I) than their isogenic diploid progenitors.

To explain the cellular mechanism of bigger leaf blades formation in tetraploid plants, we further observed changes in cross-sectional cellular characteristics of diploid and tetraploid leaf blades. The tetraploids also produced larger leaf cell lumens compared to diploids (Fig. 2E, F). The cell area and cell number of leaf veins were further measured. The adaxial epidermis cell number (Fig. 2M), xylem cell area (Fig. 2N) and number (Fig. 2O), parenchyma cell area (Fig. 2P) and number (Fig. 2Q) of leaf veins in tetraploids were significantly higher than that of diploids, indicating that the formation of larger leaf blades in tetraploid castor beans is attributed to both an increase in cell size and an increase in cell number.

In addition to alterations in leaf morphology and leaf cell size, whole-genome duplication significantly influenced phytohormone profiles. To characterize the variations in phytohormone content in plants with different ploidy levels, we measured the concentrations of major plant hormones, including IAA, GA, BR, and CTK, in diploid and tetraploid leaf tissues. The results showed that IAA and GA contents were significantly higher in tetraploids than in diploids (Fig. 2R, S). In contrast, the CTK content of tetraploids was significantly lower than that of diploids (Fig. 2T). There was no significant difference in the BR content between diploid and tetraploid plants (Fig. 2U). Different hormones often act in concert or antagonistically to regulate specific developmental processes. The reduction in CTK content might be a compensatory mechanism or part of a broader regulatory response to the increased levels of IAA and GA. The parallel trends observed in leaf size expansion and IAA accumulation suggest a potential role of auxin in mediating ploidy-dependent leaf enlargement.

### Analysis of transcriptome sequencing data

To investigate ploidy-dependent gene expression patterns, RNA-seq was performed using fresh leaf tissues from diploid and tetraploid plants. In total, 39.47 Gb of clean data was generated from six leaf samples (Table S2). The clean reads exhibited Q30 exceeding 92.41% and GC content above 43.25%, indicating a high quality of sequencing (Table S2). The clean reads were mapped to the *Ricinus communis* genome, and the efficiency of mapping ranged from 95.34 to 98.21% (Table S3).

**Fig. 3:**
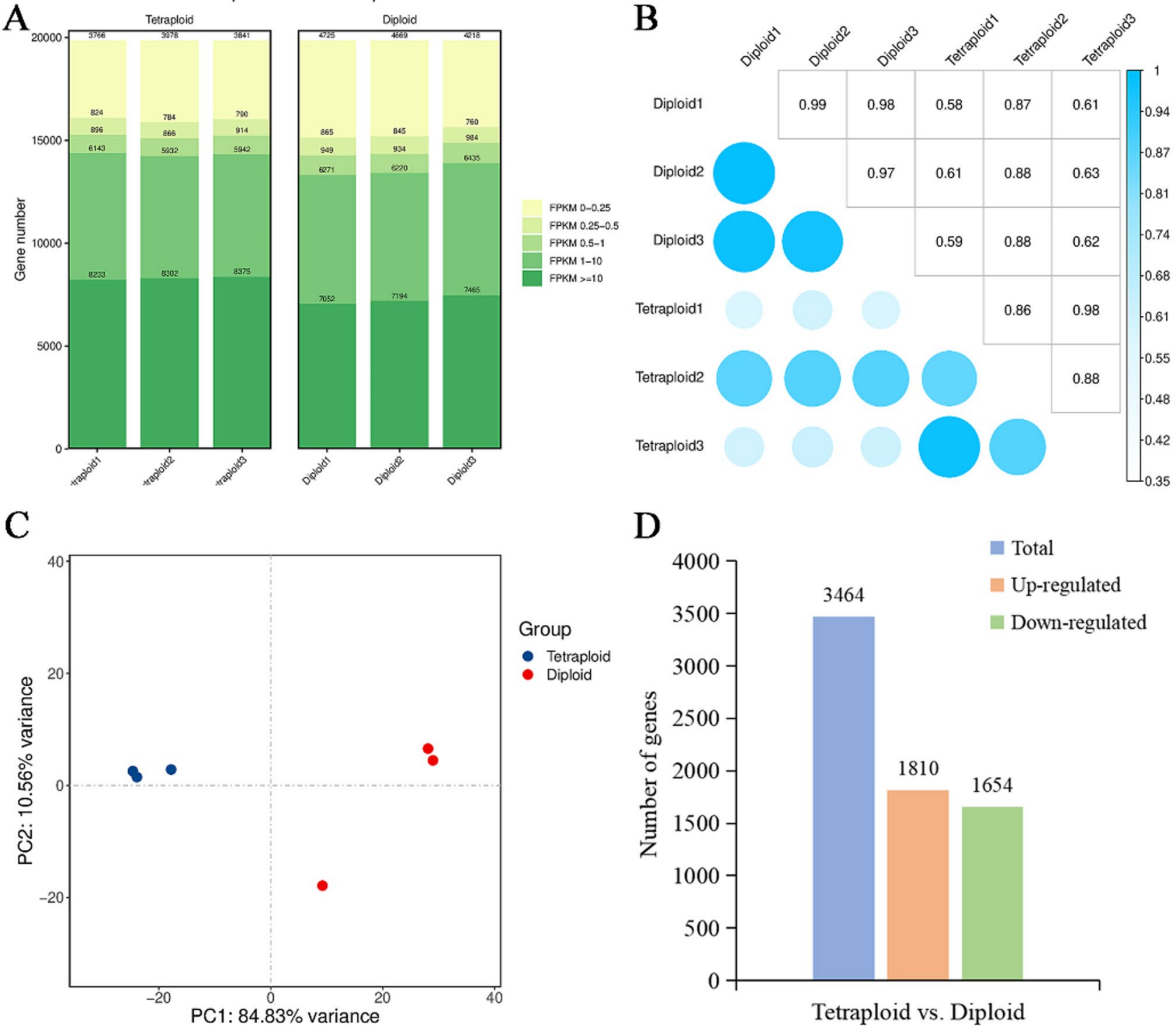
Analysis of transcriptional profiles between diploids and tetraploids. Distribution (**A**), correlation (**B**) and PCA (**C**) analysis of expression profiles from all samples. **D** Statistical analysis of up-regulated and down-regulated DEGs in different comparison groups

To analyze the reliability of the tested samples, expression levels distribution and correlation, and principal component analysis (PCA) based on the FPKM value of each library were analyzed. The results show that there was a high correlation among biological replicates, and that different ploidy levels had different gene expression patterns (Fig. 3A, B, C). These results indicated that the RNA-seq data were relatively reliable and suitable for further analysis.

**Fig. 4:**
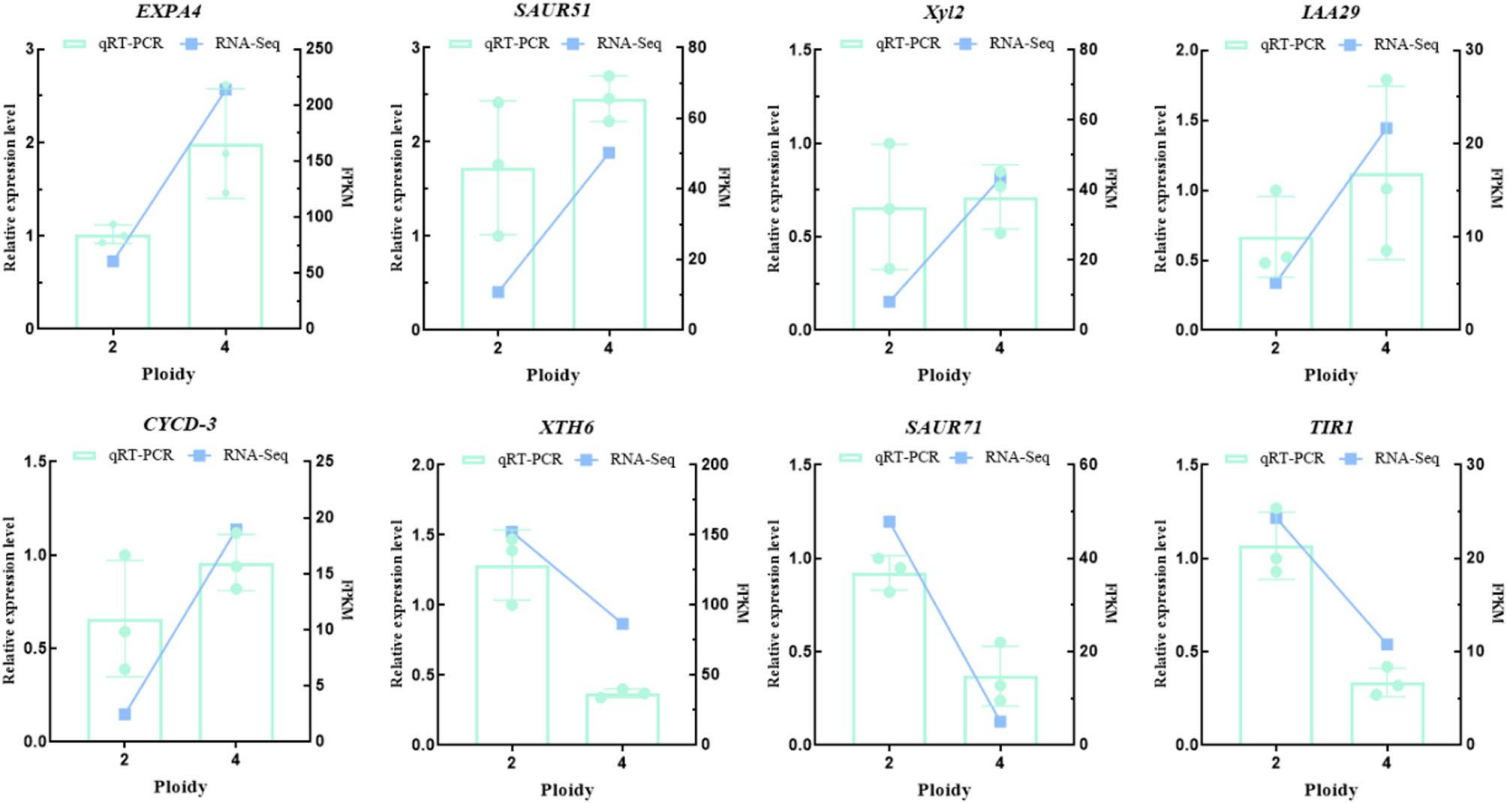
RT-qPCR verification of expression level of 8 DEGs identified by RNA sequencing. The *Y*-axis on the left indicates the relative gene expression levels analyzed by qRT-PCR, while the *Y*-axis on the right represents the FPKM value obtained by RNA-seq

To elucidate the impact of genome doubling on gene expression differences, we analyzed DEGs between diploids and tetraploids utilizing the DESeq2 software. A total of 3,464 DEGs between diploids and tetraploids were identified, including 1,810 genes up-regulated and 1,654 genes down-regulated in tetraploids relative to diploids (Fig. 3D, Table S4). To verify the availability and accuracy of the RNA-seq data, we randomly selected a total of 8 candidate genes for qRT-PCR analysis. The results demonstrated that the expression trends of these candidate genes in the qRT-PCR analysis were highly concordant with those observed in the RNA-seq analysis (Fig. 4).

### Functional enrichment analysis of DEGs

**Fig. 5:**
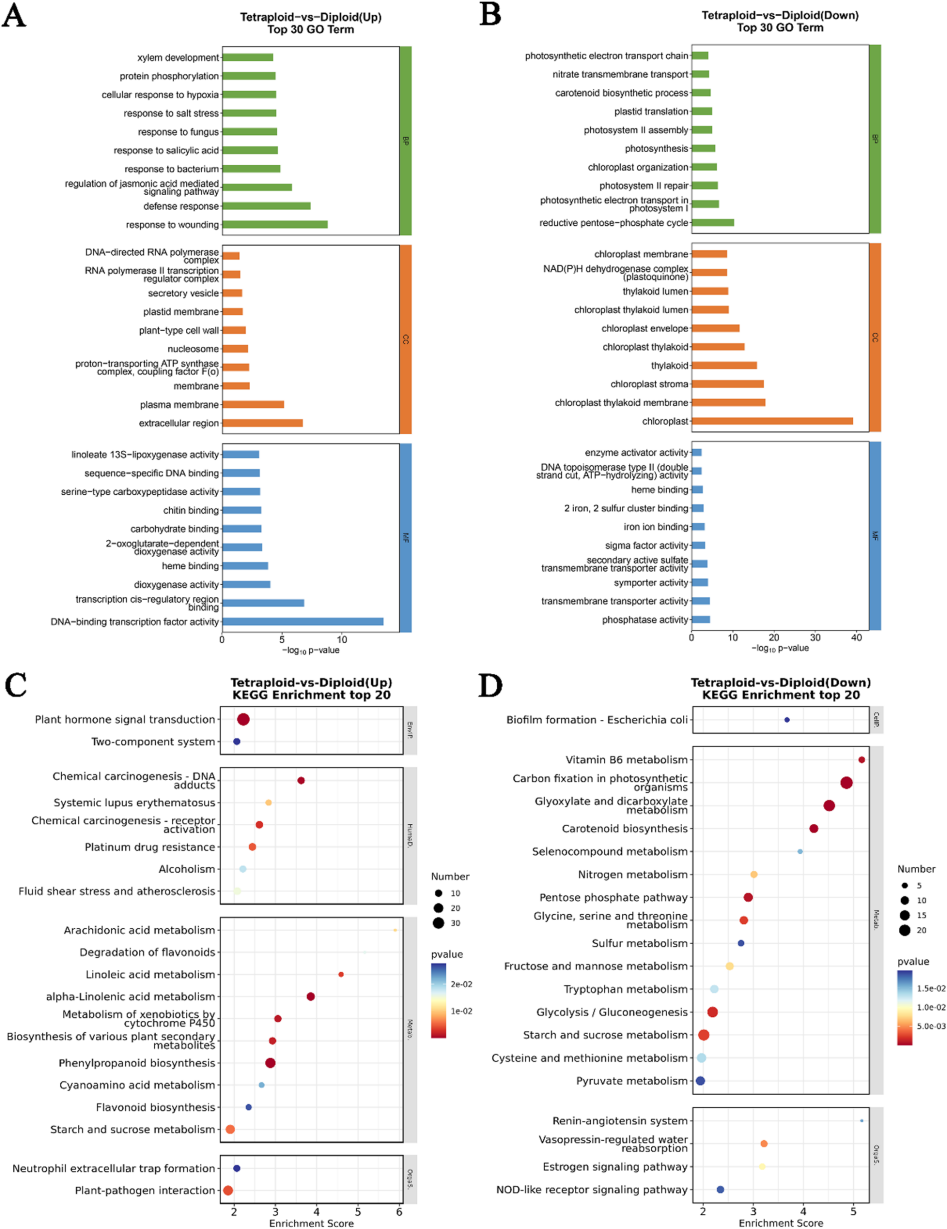
Functional enrichment analysis of DEGs between diploids and tetraploids. **A** GO enrichment analysis of up-regulated DEGs from diploids and tetraploids. **B** GO enrichment analysis of down-regulated DEGs from diploids and tetraploids. **C** KEGG enrichment analysis of up-regulated DEGs from diploids and tetraploids. **D** KEGG enrichment analysis of down-regulated DEGs from diploids and tetraploids

All DEGs between diploids and tetraploids were then subjected to enrichment analysis of Gene Ontology (GO) functions (Table S5, S6) and Kyoto Encyclopedia of Genes and Genomes (KEGG) pathways (Table S7, S8). GO enrichment analysis was conducted at three levels: biological process (BP), molecular function (MF), and cellular component (CC). The results suggested that the up-regulated genes were mainly enriched in GO terms related to DNA-binding transcription factor activity, plant-type cell wall, and xylem development (Fig. 5A). Conversely, down-regulated genes showed significant enrichment for chloroplast, photosynthetic electron transport in photosystem I, photosynthesis, and phosphatase activity (Fig. 5B).

KEGG enrichment pathway analysis revealed that up-regulated genes were predominantly associated with plant hormone signal transduction, phenylpropanoid biosynthesis, and starch and sucrose metabolism (Fig. 5C), suggesting their potential roles in leaf cell division and expansion in the tetraploid castor bean. In contrast, down-regulated genes were mainly enriched in pathways including carbon fixation in photosynthetic organisms, glyoxylate and dicarboxylate metabolism, carotenoid biosynthesis, and the pentose phosphate pathway (Fig. 5D). These enrichment results collectively indicate that ploidy-dependent differential gene expression primarily influences cellular growth processes, including metabolite biosynthesis, enzymatic activities, and cell wall dynamics.

### Identification of DEGs involved in leaf cell growth

**Fig. 6:**
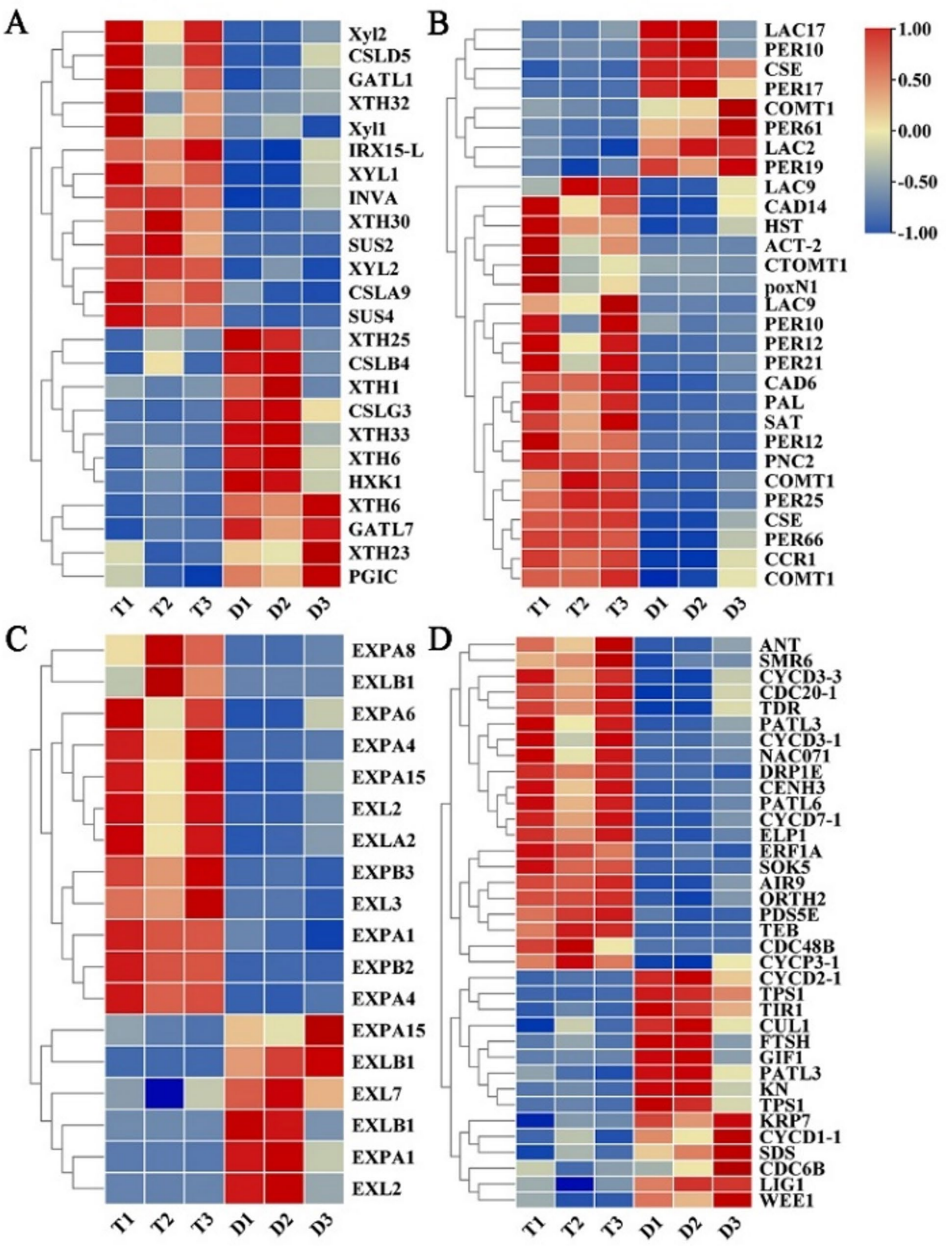
Expression heatmap of DEGs related to cell growth between diploids and tetraploids based on the FPKM value of each library. **A** DEGs related to cellulose and hemicellulose biosynthesis pathway. **B** DEGs related to lignin biosynthesis pathway. **C** DEGs related to expansins. **D** DEGs related to cell division and cell cycle. The color scale shows fragments per kilobase of transcript per million mapped reads (FPKM) values, with blue colors indicating low values and red values indicating high values

In this study, we found that the xylem and parenchyma cells in tetraploid leaf blades were significantly larger than those in diploid. In addition, cell growth, including cell-wall activities, was ongoing in diploid and tetraploid fresh leaf blades. Cell walls are fairly rigid to provide support and protection, but also extensible to allow cell growth, which is triggered by a high intracellular turgor pressure. Therefore, we identified the DEGs that encode enzymes involved in the secondary cell wall (SCW) biosynthesis pathway. In total, 5, 19, and 29 DEGs were identified as enzyme-coding genes of cellulose, hemicellulose, and lignin biosynthesis, respectively (Table S9, S10). Further expression analysis indicated that most members of these DEGs related to SCW biosynthesis were up-regulated in tetraploids relative to diploids. In the cellulose, xylan and xyloglucan hemicellulose biosynthesis pathway, the expression of 13 structural genes (including *INVA*, *SUS2*, *SUS4*, *XTH30*, *XTH32*, *CSLD5*, *CSLA9*, *IRX15*-L, *XYL1*, *Xyl2*, *XYL2*, *Xyl1*, and *GATL1*) was significantly up-regulated by 2.3- to 23.6-fold in tetraploids relative to diploids, with the expression of the *SUS4* gene particularly up-regulated by 23.6 fold. However, the expression of 11 structural genes (such as *XTH25*, *XTH23*, *XTH1*, *XTH33*, *XTH6*, *XTH6*, *CSLG3*, *CSLB4*, *GATL7*, *HXK1*, and *PGIC*) was significantly down-regulated by 1.1- to 5.2-fold in tetraploids (Fig. 6A). Unfortunately, cellulose synthases (CESAs), which were crucial for cellulose biosynthesis, have not been identified. Moreover, the expression patterns of 21 lignin biosynthesis enzyme-coding genes (such as *PAL*, *HST*, *COMT1*, *CCR1*, *CAD6*, *CAD14*, *CSE*, *LAC9*, *PER12*, *PER21*, and *PER25*) also appeared significantly up-regulated by 2.0- to 26.9- fold in tetraploids (Fig. 6B). Notably, several SCW biosynthesis genes (*SUS2*, *XYL1*, *CAD6*, *CAD14*, and *PER12*) showed particularly high expression in tetraploids (the average FPKM > 50). Consequently, these pathways and their corresponding DEGs may also be key factors affecting the growth of tetraploid cells.

Leaf blade development is the result of a series of overlapping events, including cell expansion and cell division. Expansins are also important regulators involved in cell expansion. Four expansin families are recognized in plants: α-expansin (EXPA), β-expansin (EXPB), expansin-like A (EXLA), and expansin-like B (EXLB). In our research, a total of 18 DEGs as enzyme-coding genes of expansins were significantly differentially expressed in tetraploids compared with diploids (Fig. 6C, Table S11), most of which were up-regulated (log_2_FC thresholds ranging from 1.3 to 8.2). Especially, these four expansin genes (*EXPA1*, *EXPA4*, *EXPA6*, and *EXPB3*) exhibited particularly high expression levels (the average FPKM > 50) in tetraploids, indicating they likely play significant roles in leaf cell development. Moreover, we also investigated the expression patterns of cell division and cell cycle related genes, which are crucial for the regulation of cell development. Through GO enrichment analysis, we found that a total of 36 genes were linked to cell division and cell cycle (Fig. 6D, Table S12). The process of cell division and cell cycle contained 21 up-regulated genes and 15 down-regulated genes, such as *CYCD3;1*, *CYCD3;3*, *CYCD7;1*, *CYCP3;1*, *ERF1A*, *PATL3*, *PATL6* were up-regulated by 2.0- to 30.3-fold in tetraploids. D-type cyclin (cyclin D, CYCD) participates in the regulation of cell cycle G1/S transition and plays an important role in cell division, suggesting that the increased expression of *CYCD3;1* and *CYCD3;3* in tetraploid might explain why the number of leaf cells in tetraploid was higher than in diploid. The results showed that the expression of genes related to cell expansion and cell division was higher in tetraploid leaf blades compared to diploids, resulting in enlarged tetraploid leaf blades.

### Identification of plant hormone signal transduction genes

**Fig. 7:**
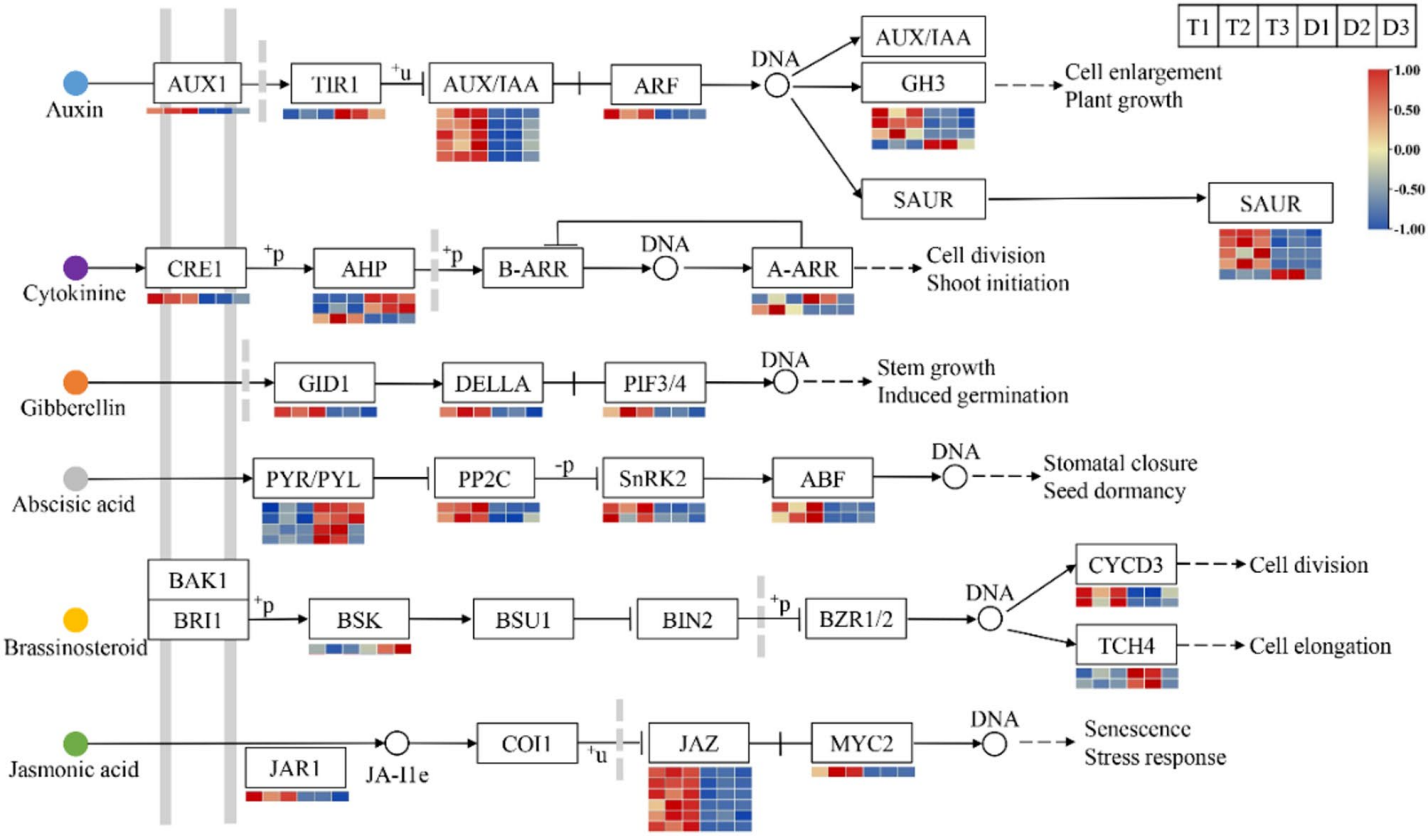
Expression heatmap of DEGs involved in the plant hormone signal transduction between diploids and tetraploids

Phytohormones play pivotal roles in regulating various aspects of plant growth and development. To elucidate the involvement of hormone signaling in leaf development, we identified 46 DEGs associated with plant hormone pathways and generated a heatmap based on their FPKM values (Fig. 7, Table S13). In the auxin signaling pathway, 14 genes showed significant up-regulation in tetraploids compared to diploids, while three genes were down-regulated. Notably, the expression levels of five IAA genes, one AUX1 gene, one ARF gene, three GH3 genes, and four SAUR genes increased by 2.1- to 84.0-fold in tetraploids. Among these, *SAUR23* and *SAUR51* demonstrated particularly high expression levels (average FPKM > 50), suggesting their potential significance in leaf cell development. Analysis of CTK signaling pathway revealed up-regulation by 1.5- to 12.1-fold of one CRE1 gene, one AHP gene, and one A-ARR gene, while one ARR4 gene and two AHP genes were down-regulated (log_2_FC thresholds ranging from − 4.5 to −1.1) in tetraploids. In the GA signaling pathway, the expression of only one GID1 gene, one DELLA gene, and one PIF3 gene was significantly up-regulated by 2.3- to 3.7-fold in tetraploids. In the BR signaling pathway, the expression of two CYCD3 genes was significantly up-regulated by 3.2- to 14.2-fold in tetraploids, and the expression of two TCH genes and one BSK gene was significantly down-regulated (log_2_FC thresholds ranging from − 3.1 to −1.8) in tetraploids. In the ABA signaling pathway, the expression of two SNRK2 genes, two ABF genes, and two PP2C genes was significantly up-regulated by 2.0- to 18.7-fold in tetraploids. However, the expression of four PYR genes was significantly down-regulated (log_2_FC thresholds ranging from − 2.7 to −1.6) in tetraploids. In the JA signaling pathway, the expression of three JAZ genes, one JAR1 gene and one MYC2 gene was up-regulated by 2.8- to 26.7-fold in tetraploids. Taken together, our results indicate that auxin-related genes might be the major regulators to promote cell growth in tetraploid leaf blades compared with diploids.

### Analysis of transcription factors and gene co-expression network

**Fig. 8:**
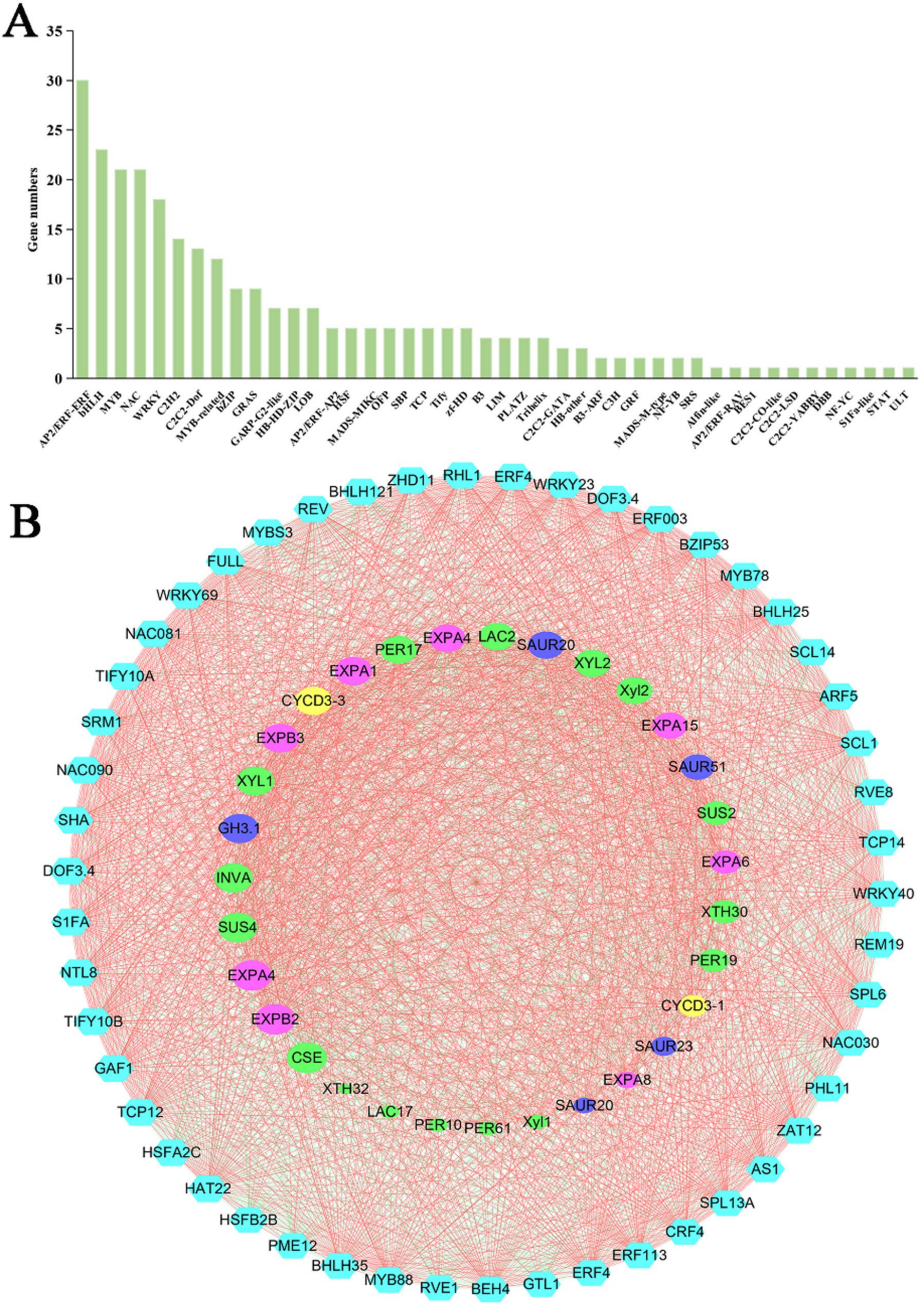
Analysis of transcription factors and gene co-expression network. **A** The distribution of differentially expressed TFs in diploids and tetraploids. **B** The gene co-expression network of TF genes and leaf development-related DEGs. Cyan hexagonal nodes indicate TFs. Circular nodes represent leaf development-related DEGs, of which green nodes indicate SWC biosynthesis structural genes, pink nodes indicate expansins, yellow nodes indicate D-type cyclin genes, blue nodes indicate auxin signaling pathway genes. The node size is positively correlated with the gene degree. The red line represents a positive correlation, while the green line represents a negative correlation. The width of the connecting line is positively related to the correlation between genes

TFs play crucial roles in regulating leaf development. To explore the roles of TFs in leaf cell development, DEGs encoding TFs in diploids and tetraploids were predicted. A total of 276 DEGs in 44 TF families were identified (Fig. 8A, Table S14), and the most common TF families observed were AP2/ERF-ERF (30, 10.9%), bHLH (23, 8.3%), MYB (21, 7.6%), NAC (21, 7.6%), WRKY (18, 6.5%), and C2H2 (14, 5.1%).

To identify TFs significantly associated with leaf development, we constructed a gene co-expression network comprising the top 50 TFs strongly correlated with 31 leaf development-related DEGs (Fig. 8B and Table S15). These TFs exhibited robust co-expression with DEGs involved in lignin biosynthesis, cellulose biosynthesis, hemicellulose biosynthesis, and cell expansion, suggesting their potential role in promoting tetraploid leaf enlargement through regulation of SCW biosynthesis and cell expansion. Within this network, *HAT22* emerged as a central hub gene with the highest connectivity. It co-expressed with the largest number of SCW biosynthesis structural genes and showed 21.4-fold up-regulation in tetraploids versus diploids. Notably, several genes—including *CSE*, *EXPB2*, *EXPA4*, *SUS4*, *INVA*, *XYL1*, *EXPB3*, *EXPA1*, and *EXPA4*—exhibited more significant interactions with TFs than other genes in the network, implying critical functions in leaf development regulation. We further identified three leaf growth-associated TFs—*SRM1*, *ERF4*, and *DOF3.4*—that were up-regulated 4.4- to 21.3-fold in tetraploid compared with diploid leaves. These likely contribute to leaf cell growth regulation. Other TFs have not yet been reported to be involved in leaf cell growth; whether they can directly or indirectly participate in leaf development remains to be studied in the future.

## Discussion

Many studies indicated that genome doubling is invariably accompanied by phenotypic variations. For example, autotetraploid *Arabidopsis* plants consistently display larger leaf areas compared to their diploid counterparts [31]. Similarly, the leaf blades of the induced autotetraploid in *Raphanus sativus* were thicker, more elliptic and crimped than diploid [52]. Wu et al. [33] documented that the autotetraploids of *P. hopeiensis* produced larger leaf blades and modified leaf blade morphology compared with diploids. Li et al. [10] also showed that the leaf length and width were significantly larger in the autotetraploids than in the diploids of *Robinia pseudoacacia*. Consistent with these findings, our study revealed that autotetraploids of castor beans produced larger leaf blades compared with diploids. Additionally, we observed significant differences in stomatal characteristics, including length and width, between induced tetraploids and diploids. This suggests that the stomatal characteristics may be used as simple and efficient indicators to distinguish plant ploidy levels [44, 45]. A similar phenomenon has been observed in tetraploid Euphorbiaceae species and oilseed crops, including *Manihot esculenta* [53], *Jatropha curcas* [54], *Hevea brasiliensis* [55] and *Arachis hypogaea* [56]. Hence, these pronounced morphological differences between diploids and tetraploids represent a promising and stable tool for plant improvement. Notably, ploidy-induced variations in stomatal size and density may significantly influence gas exchange parameters, water-use efficiency, and photosynthetic capacity in polyploid plants, suggesting the existence of unique physiological adaptations. Elucidating these adaptive mechanisms will constitute a primary research focus in our subsequent investigations.

In higher plants, the formation of organ size is directly determined by the cell size and cell number during the cell division and cell expansion processes [57]. Organ size can vary quite dramatically between different plant species, even those closely related. However, the final size of leaves and flowers within any particular plant species is remarkably uniform, suggesting a tight control over organ growth [58, 59]. A characteristic final organ size is often achieved even when cell division is disrupted due to mutations or transgenes, a phenomenon termed compensation as the increase in cell size is accompanied by a reduction in cell number [59]. However, patterns related to cell proliferation and expansion in tetraploids are different from those in diploids. An increase in DNA content, as is the case in tetraploids, generally leads to an increased cell and organ size [26]. Several previous studies have also indicated that the organ giantism of tetraploids can be attributed to the enlarged cell size and greater cell volume without a significant reduction in cell number [52, 60]. In the present study, the formation of the bigger leaf blades in tetraploid castor bean was determined by cell size and cell number according to our histological observation. This suggests that chromosome doubling may enhance cell expansion and cell division ability to create bigger leaf blades. Similar results have been reported in autopolyploid *chrysanthemum lavandulifolium* [61]. It is noteworthy that the leaf area of some octaploids was much smaller than that of diploids, and although the cell size in octaploids was larger than that in diploids [31, 44]. Therefore, a larger cell size is associated with a lower cell number in higher-ploidy plants. This result also indicated that cell size is not a unique key factor directly proportional to leaf area.

Leaf size formation is a fascinating process involving the coordinated regulation of cell division and cell expansion, which ultimately relies on the control of cell wall expansion [52]. Cell walls are fairly rigid to provide support and protection, yet they are also extensible to allow cell growth, thereby regulating the size and shape of the cell [59]. In the study, we found that the expression of most of the DEGs involved in the SCW biosynthesis pathway was significantly up-regulated in tetraploids, such as *XYL1*, *XTH32*, *XTH30*, *SUS2* and *PER12*. Among cell wall components, xyloglucans are the major hemicellulose polysaccharide in the cell walls of dicots. They can connect the cellulose microfibril surface to affect cell wall mechanical properties. The α-XYLOSIDASE1 (*XYL1*) gene encodes the only α-xylosidase acting on xyloglucans in *Arabidopsis thaliana* [62]. XTHs (xyloglucan endotransglucosylase/hydrolases) are key genes related to cell wall remodeling. XTHs achieve function by catalyzing the cleavage and reconnection process of the xyloglucan molecule and modifying the composite structure of cellulose, which can stretch cell wall and participate in the degradation and synthesis of the plant cell wall [63]. Previous studies have also shown that the xyloglucan/cellulose composite structure makes the cell wall more elastic than simple cellulose structure [64]. Furthermore, XTHs were proven to slacken the cell wall, leading to cell growth and elongation [63]. This suggests that flabby cell walls may reduce the restrictions of protoplast expansion and lead to an increase in cell volume thereby increasing the volume and area of organs.

Expansins are an important superfamily of cell wall loosening proteins involved in plant cell expansion and elongation, which can non-enzymatically trigger the relaxation of the cell wall for expansion [65]. The members of the EXPA and EXPB subfamilies act on a variety of plant growth and developmental processes relating to cell wall modification, such as leaf formation, elongation, and shape [66]. In a previous study, the tobacco plant genes *NtEXPA1* and *NtEXPA4* encode the EXPA proteins involved in the regulation of cell growth and extension [67]. Overexpression of *NtEXPA1* resulted in an increased size of tobacco leaves and stems because of the larger size of the individual cells [67]. Furthermore, overexpressing the *BrEXPA1* gene promoted leaf growth in *Arabidopsis thaliana* [68]. Zhu et al. [69] reported that the increased expression of *EXPA1*, *EXPA4*, and *EXPA6* genes might contribute to the increased leaf cell size in tetraploid *Actinidia chinensis*, potentially by enhancing the cell wall extensibility. Qiao et al. [70] also found that chromosome doubling resulted in the up-regulation of the *EXPB3* gene, which is involved in cell growth and differentiation in tetraploid *Dendrocalamus latiflorus*. Expansion gene family members *EXPA1*, *EXPA4*, *EXPA6*, and *EXPB3* were up-regulated in tetraploids in the current study, which may lead to cell growth in tetraploid leaf blades. Similar results have been reported in previous studies [69, 70].

CYCD3 plays a crucial role in determining cell number in developing lateral plant organs by regulating the G1/S transition, and it contributes to the transition from cell production to cell expansion in these organisms [71]. For example, overexpression of *CYCD3*;*1* in *Arabidopsis* stimulated cell division and increased the cell number by controlling the length of the mitotic window [72]. Overexpression of *PtoCYCD3*;*3* significantly facilitated the division of leaf adaxial epidermal cells and palisade tissue cells, thus promoting the leaf enlargement and vegetative growth of *Populus* [73]. In addition, we found that the expression of two CYCD3 (*CYCD3*; *1* and *CYCD3*; *3*) genes was significantly up-regulated in tetraploids. These results demonstrated that a higher activity of cell division significantly increased total cell numbers, and a greater cell number contributed to the larger leaf blade in tetraploids.

Auxin is a core regulator of plant growth and development and an effective promoter of cell division and cell expansion [74]. Additionally, auxin is involved in the formation of the cell wall, which can greatly enhance the cell wall flexibility and plasticity [52]. Three major classes of auxin-responsive transcription factors controlled by the Auxin/indole-3-acetic acid (AUX/IAA), Gretchen Hagen 3 (GH3), and small auxin up RNA (SAUR) genes regulate auxin signaling [74, 75]. SAUR, the largest family of early auxin response genes, plays crucial roles in multiple processes, including cell expansion, leaf growth and senescence, auxin transport, tropic growth and so on [75]. Previous studies have shown that the SAUR19–24 functions as positive effectors of cell expansion in *Arabidopsis thaliana* [76]. *SAUR51* was specifically expressed in the expanded leaves of *Arabidopsis thaliana*, and overexpression of *SAUR51* showed a distinct cell elongation phenotype [77]. In our study, the expression of two *SAUR20*, one *SAUR23*, and one *SAUR51* genes was significantly up-regulated in tetraploids, and this likely contributed to leaf cell expansion.

Leaf growth requires coordinated regulation by both structural genes and TFs. Homeodomain-leucine zipper (HD-Zip) II TFs regulate diverse plant biological processes. In this study, *HAT22*, which is predicted to be an ortholog of *Populus trichocarpa PtrHAT*22, was found to be highly co-expressed with enzyme-coding genes involved in cell wall biosynthesis and cell expansion. Overexpression of *PtrHAT22* resulted in significant decreases in lignin content, cellulose content, and SCW thickness, while increasing hemicellulose content in *P. trichocarpa* [78]. Similarly, overexpressing *EcHB1*, the ortholog of *HAT22* in *Eucalyptus camaldulensis*, shows decreased acid-soluble lignin in transgenic tobacco [79]. *HAT22* also regulates proliferation and differentiation in the wood-forming cambium cells of *Salix suchowensis* [80]. Therefore, *HAT22* may regulate tetraploid leaf cell expansion by modulating SCW biosynthesis.

As one of the largest gene families in plants, MYB TFs are involved in multiple biological processes. Among the family members, a class of *SRM1* proteins belonging to the MYB family plays a role in leaf development. Loss-of-function of the *SRM1* gene changes the morphology of rosette leaves in *Arabidopsis* and makes the leaves smaller, while the overexpression of the *SRM1* gene promotes the vegetative growth of the leaves [81]. Previous studies have shown that silencing of *CaSRM1* caused the development of uneven leaf margins and curling of pepper leaves [82]. In tomato, SlSRM1-like affects leaf development and is expressed in multiple tissues, and its expression is induced by auxin [83]. ERF4 promotes endoreduplication and cell growth as a positive regulator. *Arabidopsis* plants overexpressing *ERF4* develop larger cells and organs, while *erf4* mutants display smaller leaves, petals, and seeds than wild-type [84]. *DOF3.4* drives root cell division in *Arabidopsis* through activation of cyclin *CYCD3;3*, promoting radial cell elongation [85]. In this study, *SRM1*, *ERF4*, and *DOF3.4* showed high co-expression with genes regulating leaf cell development. While these findings suggest potential regulatory roles, functional validation of their impact on leaf morphogenesis requires further investigation.

Accordingly, it can be inferred that the interactions among DEGs related to cell wall construction, cell expansion, cell division, and auxin might be the primary factors contributing to the enlargement of tetraploid leaves, a finding that has not been previously reported. However, further functional validation is needed. Interestingly, while tetraploids exhibited larger leaf blades with greater cell numbers and size, the genes associated with photosynthesis and carotenoid biosynthesis were down-regulated. This apparent paradox may reflect metabolic prioritization during active leaf expansion, where resources are allocated toward cell growth rather than photosynthetic capacity per cell. Larger leaves require more energy and nutrients for their maintenance and growth, which could potentially come at the expense of photosynthetic efficiency. Similar patterns have been observed in other polyploid systems, where whole-organ photosynthesis compensates for reduced cellular-level efficiency [59, 86]. The temporal dynamics of this regulation warrant further investigation across leaf developmental stages.

## Conclusion

**Fig. 9:**
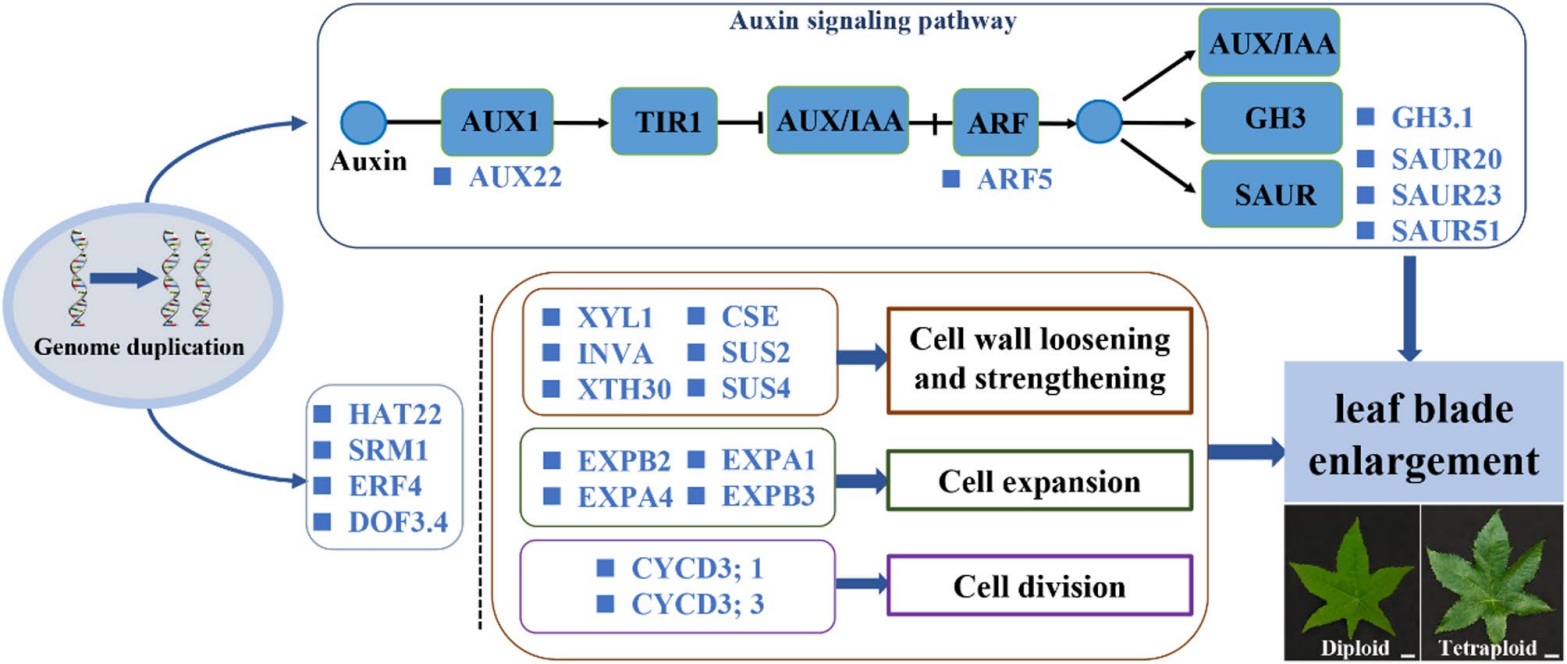
Putative model of the internal mechanisms of bigger leaf formation in castor bean after chromosomal doubling. *Bar* 2.0 cm

After a whole-genome duplication event, tetraploids of castor beans produced larger leaf blades compared with diploids. The formation of larger leaf blades in tetraploid castor beans is attributed to both an increase in cell size and an increase in cell number according to our histological observation. The expression of key genes related to cell wall loosening and strengthening during the SCW biosynthesis process was significantly up-regulated in tetraploids, such as *SUS2*, *SUS4*, *XYL1*, *Xyl2*, *XTH30*, and *XTH32*, which potentially contribute to leaf cell growth and elongation. The significant increase in both cell size and number in tetraploid leaf blades was attributed to the up-regulated expression of genes related to cell expansion and cell division in tetraploids, including *EXPA1*, *EXPA4*, *EXPA6*, *EXPB3*, *CYCD3*;*1* and *CYCD*3;3. Concurrently, auxin-responsive genes (*SAUR20*, *SAUR23*, and *SAUR51*) in the auxin signaling pathway showed significant up-regulated in tetraploids, facilitating leaf cell expansion. TFs including *HAT22*, *SRM1*, *ERF4*, and *DOF3.4* likely regulate cell expansion and elongation pathways, ultimately driving tetraploid leaf blade enlargement (Fig. 9). This study might provide new insight into understanding the potential molecular mechanisms of bigger leaf blades formation for autopolyploid plants.

## Supplementary Information

Supplementary Material 1.

## Data Availability

The raw sequence data reported in this paper have been deposited in the Genome Sequence Archive in National Genomics Data Center [87, 88], China National Center for Bioinformation / Beijing Institute of Genomics, Chinese Academy of Sciences (GSA: CRA023330) that are publicly accessible at https://ngdc.cncb.ac.cn/gsa.

## Abbreviations

IAA: Indole-3-acetic acid
GA: Gibberellins
BR: Brassinosteroids
CTK: Cytokinin
FPKM: Fragments per kilobase of transcript per million fragments mapped reads
qRT-PCR: quantitative real-time polymerase chain reaction
DEGs: Differentially expressed genes
GO: Gene Ontology
KEGG: Kyoto Encyclopedia of Genes and Genomes
TFs: Transcription factors
SCW: Secondary cell wall
PCA: Principal component analysis
